# Oral, frozen fecal microbiota transplant (FMT) capsules for recurrent *Clostridium difficile* infection

**DOI:** 10.1186/s12916-016-0680-9

**Published:** 2016-09-09

**Authors:** Ilan Youngster, Jasmin Mahabamunuge, Hannah K. Systrom, Jenny Sauk, Hamed Khalili, Joanne Levin, Jess L. Kaplan, Elizabeth L. Hohmann

**Affiliations:** 1 Divisions of Infectious Diseases, Massachusetts General Hospital, Boston, MA USA; 2 Division of Gastroenterology, Massachusetts General Hospital, Boston, MA USA; 3 Clinical and Translational Epidemiology Unit, Massachusetts General Hospital, Boston, MA USA; 4 Division of Pediatric Gastroenterology, Massachusetts General Hospital for Children, Boston, MA USA; 5 Cooley Dickinson Hospital, Northampton, MA USA; 6 Division of Infectious Diseases, Boston Children’s Hospital, 300 Longood Ave, Boston, MA 02115 USA; 7 Harvard Medical School, Boston, MA USA

**Keywords:** Fecal microbiota transplant, *Clostridium difficile*, Microbiome, Oral administration

## Abstract

**Background:**

Fecal microbiota transplantation (FMT) has been shown to be safe and effective in treating refractory or relapsing *C. difficile* infection (CDI), but its use has been limited by practical barriers. We recently reported a small preliminary feasibility study using orally administered frozen fecal capsules. Following these early results, we now report our clinical experience in a large cohort with structured follow-up.

**Methods:**

We prospectively followed a cohort of patients with recurrent or refractory CDI who were treated with frozen, encapsulated FMT at our institution. The primary endpoint was defined as clinical resolution whilst off antibiotics for CDI at 8 weeks after last capsule ingestion. Safety was defined as any FMT-related adverse event grade 2 or above.

**Results:**

Overall, 180 patients aged 7–95 years with a minimal follow-up of 8 weeks were included in the analysis. CDI resolved in 82 % of patients after a single treatment, rising to a 91 % cure rate with two treatments. Three adverse events Grade 2 or above, deemed related or possibly related to FMT, were observed.

**Conclusions:**

We confirm the effectiveness and safety of oral administration of frozen encapsulated fecal material, prepared from unrelated donors, in treating recurrent CDI. Randomized studies and FMT registries are still needed to ascertain long-term safety.

## Background

The epidemiology of *Clostridium difficile* infection (CDI) is evolving. Rates of infection are increasing and response to standard antimicrobial treatment with metronidazole or vancomycin may be suboptimal [1, 2]. Fidaxomicin has been shown to reduce the rate of recurrence compared to vancomycin; however, it has not been studied extensively in patients with multiple recurrences, and use is limited by cost [3]. Fecal microbiota transplant (FMT) has been shown to be safe and effective in treating refractory or relapsing CDI [4–8], but its use has been limited by practical barriers. Among other concerns, the administration of FMT by colonoscope or naso-gastric/duodenal tube exposes the patient to some risk and discomfort. We recently reported a preliminary feasibility study using orally administered frozen fecal capsules, prepared from unrelated donors, to treat 20 patients with recurrent CDI [9]. Following these encouraging results, we have continued treating patients with FMT capsules. We report our clinical experience in a large cohort with structured follow-up.

## Methods

At the time of writing, 202 patients had been treated with FMT capsules at Massachusetts General Hospital. The outcomes of 180 patients who had completed an 8 week follow-up assessment (with 154 completing follow-up/reaching 6 months) are reported herein. Patients over the age of 7 years, with three or more mild-to-moderate episodes of CDI or two episodes requiring hospitalization, were offered capsule FMT. Absolute exclusions were neutropenia (absolute neutrophil count < 500) and prednisone > 40 mg/day; other immuno-suppressed patients were considered with agreement of referring physicians. The study was reviewed and approved by the Partners Human Research Committee Institutional Review Board and submitted to the Food and Drugs Administration (IND 16011, sponsor E. Hohmann MD). Recipients or parents provided written consent, which discussed the risks, benefits, and investigational nature of the procedure. Children provided assent. Donor screening, preparation of the frozen capsules, FMT procedures, and patient follow-up were unchanged from the previous report [9]. Briefly, donors were healthy adults with a normal body mass index, passed the American Association of Blood Banks donor questionnaire, and underwent physical examination and extensive laboratory testing for general health and infectious diseases as described [9].

Donated fecal matter was blenderized, sieved, centrifuged, and suspended in concentrated form in sterile saline with 10 % glycerol. The suspension was double-encapsulated in hypromellose capsules (Capsugel, Cambridge, MA) and stored at –80 °C for up to 6 months pending use. Processing was done entirely under ambient air. FMT recipients discontinued any anti-CDI treatment for 24–48 hours prior to FMT, and were given 15 capsules on each of two consecutive days with water or apple sauce. The 30 capsules contained sieved, concentrated material derived from a mean of 48 g of fecal matter. Recipients were nil per os 4 hours before and 1 hour after dosing. Those with worsening diarrheal symptoms at least 72 hours after the second dosing day had fecal samples retested for *C. difficile*. If they had diarrhea and still tested positive, they were retreated with 4–14 days of standard antibiotic therapy for CDI and were offered another FMT. Patients were followed on a standardized schedule by phone (3–5 days, 10–14 days, 2 months, and 6 months after FMT) with structured questionnaires recording stool frequency, general and gastrointestinal well-being, and mild, moderate, or severe adverse events (grades 1, 2, or 3 based upon CTCAE V.4.0). The primary endpoint was defined as clinical resolution/no relapse of diarrhea whilst off antibiotics for CDI at 8 weeks after last capsule ingestion and safety, defined as any FMT-related adverse events grade 2 or above. Resolution of diarrhea was defined as three or fewer bowel movements per 24 hours. For brevity going forward we have defined this resolution as a “cure” though we appreciate “resolution of symptoms/no relapse” may be preferred. Fecal microbiome analyses were beyond the scope of this treatment study.

## Results

**Fig. 1:**
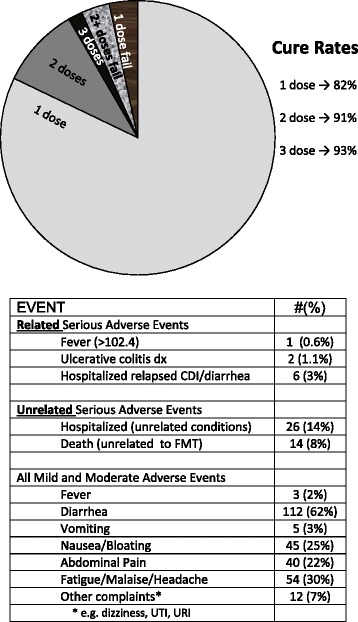
Cure rates and cumulative adverse events in 180 capsule fecal microbiota transplant recipients. All recipients were followed for 8 weeks, and 154 completed 6 months of follow-up

From July 2013 through April 2016, 180 patients aged 7–95 years (median 64) were treated using fecal material collected from seven donors; 82 recipients were over 65 years old, whereas five were < 18 years and 15 were > 85 years. Cure rates and adverse events (to 6 months) are shown in Fig. 1. Of the 180 patients reaching 8 weeks, 147 were cured of CDI after the first administration of fecal capsules (82 %). Twenty six individuals relapsed within 8 weeks and were re-treated, with 17 responding, resulting in an overall cure rate of 91 % with one or two treatments. Six individuals declined re-treatment (our standard procedure in these cases is to offer long-term suppressive oral vancomycin treatment). Three patients were cured after a third administration, but were considered “non-responders” as per protocol definition. One patient received three treatments, relapsed, and was advised to continue suppressive vancomycin.

Five patients relapsed in the 2–6 month window, one due to antibiotic treatment, one due to chemotherapy, and three spontaneously. Four patients were retreated and one was lost to follow-up. Of the four patients retreated, all durably resolved, but one died of recurrent cancer.

Only three serious adverse events Grade 2 or above (one transient high fever, two new endoscopic diagnoses of ulcerative colitis) deemed related or possibly related to FMT were observed. Two patients who had ongoing, worsening diarrheal symptoms after FMT were found by colonoscopy with biopsy to have ulcerative colitis, which was previously suspected in one. Fourteen patients died within the 6 month follow-up window; no deaths were deemed related to FMT by either the FMT team or by referring/treating physicians. Six patients (3 %) were re-hospitalized for relapsed CDI; other hospitalizations were for other/underlying conditions. Two patients experienced small bowel obstructions after FMT. One was admitted with a partial small bowel obstruction 8 weeks after FMT (his fifth episode of bowel obstruction in the past 18 months). This resolved with conservative management and was believed related to past abdominal surgeries. Another patient with rheumatological disease and past immunomodulatory therapy had a small bowel obstruction 4 weeks and 12 weeks after two FMT treatments, respectively. This patient required surgery, which revealed a chronic inflammatory mass, attributed by the surgeon to complications of an adhesion or an umbilical hernia which led to perforation and contained abscess formation. A single patient with a history of emesis after taking oral medications vomited up 15 intact capsules immediately after ingestion of his second day’s dose, but was cured of CDI. Five patients experienced vomiting 6 to 9 hours after the initial 15 capsules, and did not bring up any capsules – all but one of these five took a second treatment with 15 capsules, did not vomit, and had resolved CDI; one declined and relapsed. Four patients had transient low grade fever. Mild transient gastrointestinal adverse events included post-treatment diarrhea, nausea, abdominal discomfort and bloating, and were reported in the majority of patients (Fig. 1). Seven patients relapsed with CDI between 8 weeks and 6 months, four without obvious precipitant, one each possibly related to antibiotics, chemotherapy, or prednisone treatments. There was no documented or suspected transmission of infection. Three patients on suppressive oral antibiotics (ciprofloxacin, nitrofurantoin, trimethoprim/sulfamethoxazole) that were deemed necessary by their infectious disease physicians, and viewed as exclusionary by other FMT providers, were treated with FMT while on these drugs, and all were cured of CDI at 8 weeks.

## Discussion

Herein, we confirm the effectiveness and safety of oral administration of frozen encapsulated fecal material, from unrelated donors, in treating recurrent CDI, with an overall “cure” rate, defined as resolution/no relapse of diarrhea at 8 weeks, of 91 % with one or two administrations of 30 capsules. This compares favorably to rates reported for colonoscopic administration, which are in the range of 93 to 96 % [5]. Flare or unmasking of inflammatory bowel disease is a reported complication of both FMT and CDI, and occurred in two patients here. The majority of patients reported mild gastrointestinal complaints after FMT, which resolved without intervention. We use a standardized grading system for adverse events, and about 84 % of reported events were Grade 1 (symptoms causing no or minimal interference with usual social and functional activities). Death and hospitalization rates reflect the co-morbidities of this patient population. Recent case reports have highlighted rare adverse outcomes after FMT [10, 11]. In both reports, it seems the invasive procedure performed to administer the inoculum may have been a contributing factor. The use of capsules obviates the need for any invasive procedures, potentially overcoming procedure-associated risks.

FMT has been shown to be the most cost-effective treatment strategy for recurrent CDI, when compared to vancomycin or fidaxomicin [12]. Greatly simplifying delivery and reducing the person-power and infrastructure required, oral administration is likely to make FMT even more favorable in terms of cost–benefit. Resolution of recurrent CDI in patients in acute and long-term care facilities may confer an additional health system and societal benefit by reducing transmission. Nevertheless, some potential limitations of oral administration of FMT should be kept in mind. The use of capsules requires the patients’ cooperation and is limited in patients with dysphagia or gastrointestinal dysmotility. Vomiting and aspiration remain a potential concern, though in our experience, it is a rare occurrence. While the administration is greatly simplified, preparation of capsules is labor-intensive compared to inocula intended for endoscopic administration, and might not be feasible in some clinical settings.

The main strength of this study lies in our sample size and structured follow-up. We acknowledge a lack of placebo or active comparator; we note that placebo administration is challenging in patients with multiple episodes of CDI or hospital admissions. Reasons for relapse after FMT would be interesting to characterize from a microbiome perspective.

## Conclusion

Capsule FMT is now offered as standard care for recurrent or refractory CDI at Massachusetts General Hospital. Nevertheless, long-term safety concerns remain an important issue, especially in younger patients. Transmission of infections and complications related to microbiome manipulation are also potential concerns. Therefore, larger studies and FMT registries will be of value.

## Abbreviations

CDI: *Clostridium difficile* infection
FMT: fecal microbiota transplant
